# Data driven algorithmic advances in defect detection of additively manufactured materials using non-destructive testing: a review

**DOI:** 10.3389/frai.2026.1914473

**Published:** 2026-09-15

**Authors:** Smita Pawar, Ramachandra Pujeri, Sachin Pawar, Suvarna Pawar, Daria Derusova

**Affiliations:** 1 Department of Computer Science and Engineering, MIT School of Computing, MIT Art, Design and Technology University, Pune, India; 2 Department of Mechanical Engineering, MIT School of Engineering and Sciences, MIT Art, Design and Technology University, Pune, India; 3 School of Non-destructive Testing, Tomsk Polytechnic University, Tomsk, Russia

**Keywords:** additive manufacturing (AM), data-driven, deep learning, defect detection, machine learning, non-destructive testing (NDT), physics-informed ML

## Abstract

With the fifth industrial revolution, additive manufacturing (AM) is being extensively employed in industries including manufacturing, automotive, aerospace, medical, and energy. It is now accepted as a state-of-the-art and innovative way of producing complex parts that can exhibit enhanced mechanical properties and fewer design constraints. However, AM creates unique challenges in maintaining the quality and reliability of manufactured components. Addressing the detection and classification of defects induced by AM processes including cracking, porosity, lack of fusion, delamination and surface irregularities, during and after manufacturing is therefore critical. Traditional non-destructive testing (NDT) techniques including ultrasound testing, X-ray computed tomography (XCT), optical or microscopy based methods and thermography, provide reliable defect detection but are often slow, operator dependent, and poorly suited to *in-situ*, layer-by-layer monitoring. Over the past decade, the consolidation of data-driven i.e., machine learning (ML) and deep learning (DL) models with these NDT modalities have emerged as a promising route toward faster, more automated, real-time *in-situ* monitoring and control, predictive analytics for maintenance and qualification decisions. This review synthesizes 50 studies spanning thermographic, ultrasonic, acoustic, optical, and computed tomography-based NDT approaches combined with ML models ranging from convolutional neural networks (CNNs) to physics-informed learning frameworks. A structured methodology, classification table, and comparative performance analysis of ML algorithms are presented, followed by a discussion of advantages, limitations, and open research gaps. This review finds that thermography-based approaches dominate the literature, the CNN-based deep learning models generally report the highest classification accuracies (90%−99%), and physics-informed and hybrid ML approaches offer improved generalization for process control. The challenges include limited standardized datasets, inconsistent qualification frameworks and the computational demands of real-time deployment. This review concludes with recommendations for standardized benchmark datasets, multi-modal fusion, physics-informed and explainable AI models, and real-time closed-loop process control, all aimed at advancing data-driven NDT from laboratory demonstration toward industrial qualification of additively manufactured components.

## 1 Introduction

Additive manufacturing (AM), often known as 3D-printing builds components layer-by-layer from models, in contrast to subtractive manufacturing processes (ASTM Standard F2792, 2012). This layered approach makes the manufacturing of geometrically complex and lightweight structures possible which can be difficult with conventional manufacturing methods. Over the past decade, AM has moved beyond prototyping into direct production of end-use parts in aerospace, automative, medical sectors (Karkun and Dharmalingam, 2022). Despite these advantages, AM processes have significant impact of process parameters like laser power, scan speed, layer thickness, power quality, and thermal history. Various defects can be introduced by small variations in these parameters, many of which are not visible externally and can only be detected through non-destructive testing (AbouelNour and Gupta, 2022; Everton et al., 2016). Conventional NDT techniques such as radiography, ultrasonic testing (UT), and computed tomography (CT) have long been used in manufacturing quality assurance, but their application to AM introduces new challenges related to complex internal geometries, rough surfaces and the need for *in-situ* layer-by-layer monitoring rather than solely post-process inspection (Lopez et al., 2018; Villarraga-Gómez et al., 2018; Davis et al., 2019). This has motivated the development of thermographic, acoustic emission, and optical monitoring techniques tailored to the AM process, often applied during manufacturing itself (Baumgartl et al., 2020; Pandiyan et al., 2021; Chen et al., 2023). Simultaneously, the growing amount and complexity of sensor data produced during AM process has prompted the usage of machine learning (ML) or deep learning (DL) models to automate detecting defects which enables process control in real-time and supports predictive maintenance decisions (Bai et al., 2025; Saberironaghi et al., 2023; Deshpande et al., 2024).

### 1.1 Background on additive manufacturing

Additive Manufacturing is described as a “process of joining materials layer by layer using 3D model data to make objects” by American Society for Testing and Materials (ASTM) standard F2792 (ASTM Standard F2792, 2012). It is advantageous to use additive manufacturing to reduce manufacturing time without compromising the quality of printed objects. Significant advancements are being made in the production of various materials and finished goods through additive manufacturing. For instance, Karkun and Dharmalingam (2022) provided an outline of the technologies used in 3D printing and their aerospace applications in one of the review papers. AM forms physical objects using various materials including plastics, metals, and composites by combining powder, sheet materials or liquid layer by layer. Among various technologies, binder jetting, stereolithography, Fused Deposition Modeling/ Filament Fused Fabrication (FDM/FFF), directed Energy Deposition (DED), Selective Laser Melting (SLM), and Powder Bed fusion (PBF) are few AM technologies that are applied to prototyping, customized parts manufacturing, and complex geometries (Karkun and Dharmalingam, 2022).

### 1.2 Types of defects

Several factors can lead to defects in an AM component. Two types of defects can be found in AM parts: internal or subsurface defects and surface defects. Among all, delamination, stringing, warping, and balling are regarded as surface defects because they are visible on the outer edge or top layer of a printed part whereas voids, pores, and keyhole porosity, which appear on the part's surface, subsurface, or interior are referred as subsurface defects (Baumgartl et al., 2020; Szymanik et al., 2021; Paraskevoudis et al., 2020; Zhou et al., 2024; Chicote et al., 2022; AbouelNour and Gupta, 2022). Furthermore, voids and keyhole porosity are exclusive to metal-based AM processes like PBF or DED, whereas delamination and warping are unique to FFF/FDM procedures (Borish et al., 2020).

### 1.3 Importance of defect detection in AM materials

Although AM is increasingly employed in various industries, especially aerospace, automotive, and medical, the application of accurate defect detection techniques to guarantee the integrity and performance of materials in these applications is crucial (Chen et al., 2022; Seifi et al., 2016). Similarly, along with the development of AM technologies, the defect detection technique in AM is one of the critical challenges, where safety and reliability are significant. Moreover, the defect detection methodology in AM tends to rely on the size, shape, and morphology rather than their origin (ASTM International, ISO/ASTM TR 52905, 2023). Identification of material defects is essential for both user safety and regulatory standards as well. To address this issue, non-destructive testing (NDT) techniques and machine learning (ML) can be used. It defines an area for the development and facilitates continuous improvement during and after production.

### 1.4 Non-destructive testing techniques overview

Non-destructive testing (NDT) techniques, as the word indicates, are used to detect internal defects in objects without any physical contact. The integration of NDT and AM is challenging due to complex geometry of parts, uneven surface and deep anomalies (Lopez et al., 2018). However, NDT techniques for AM products evaluate dimensional accuracy, surface quality, internal structures and defect identification (Szymanik, 2022). In AM materials, the most widely used methods for detecting surface flaws are microscopy (Grimm et al., 2015; Wang et al., 2018), laser profilometry (Borish et al., 2020) and vision (Paraskevoudis et al., 2020; Zhou et al., 2024; Okarma and Fastowicz, 2019). On the other hand, computed tomography (CT) (Chen et al., 2021; Acharya et al., 2022; Villarraga-Gómez et al., 2018), ultrasonic testing (Davis et al., 2019; Chicote et al., 2022; Lopez et al., 2018), thermography (Baumgartl et al., 2020; Szymanik et al., 2021; Rodríguez-Martín et al., 2022; Szymanik, 2022; Boiko et al., 2022; Newcome, 2021; Heifetz et al., 2020) and acoustic emission (Pandiyan et al., 2021; Chen et al., 2023) are the primary methods used to assess the internal structure of objects, including defects. Moreover, more recent work has explored electromechanical impedance and modal analysis in combination with X-ray inspection for additively manufactured plastics, providing a comparative evaluation of complementary NDT modalities for structural assessment (Çakir et al., 2026).

Among all, thermography is a powerful NDE method to detect subsurface or internal defects by examining thermal images. Since it boosts the capacity to examine deeper layers inside the evaluated part and produces a stronger thermal contrast, active thermography generally performs better than passive method. This method is mostly used to identify and measure the defects in different materials such as metallic (Cerniglia and Montinaro, 2018; Dong et al., 2020), polymeric (Lahiri et al., 2012), and concrete ones (Mac et al., 2020). Over the past decade, active thermography has found greater application in the detection of defects that are either naturally occurring or artificially introduced in AM components, particularly PBF processed (Heifetz et al., 2020; Montinaro et al., 2018).

### 1.5 Machine learning in defect detection

Due to the constraints of NDT techniques in the context of complex AM structures, there was a need to develop robust, automated, and high-precision methodologies that uses the power of data-driven algorithms to process and interpret the data produced by these methods. Therefore, machine learning (ML) and deep learning algorithms (DL) were integrated with NDT techniques to improve the accuracy and speed of defect detection besides being cost effective. The neural networks, particularly the Convolutional Neural Networks (CNNs) have proved to be highly promising as far as the development of more effective and efficient detection of AM materials is concerned. The applications of machine learning (ML) models to NDT in AM can be classified into the following categories:

(i) *Automated Defect Detection*: ML algorithms, particularly CNNS, are used to analyze images and signals from various NDT methods (e.g., XCT, ultrasonic testing, thermography) for identifying defects including porosity, cracks, and lack of fusion. This kind of automation minimizes the human error and accelerates the visual inspection.
(ii) *Real-Time Process Monitoring and Control*: AI systems are applied along with the on-site sensors including thermal cameras or acoustic emission sensors to monitor the layer-by-layer AM process. ML Models respond to this real time information to identify an abnormality and may even lead to adaptive control over process parameters such as scan speed and laser power preventing the production defects.
(iii) *Predictive Analysis and Maintenance*: The ML models are trained to predict possible material or component behavior and forecast failures using the data collected from NDT techniques. A distinct body of work applies physics-informed learning to AM defect and quality prediction, integrating known physical laws including heat transfer, melt pool dynamics into the ML architectures.

Figure 1 brings one such example of a material defect testing system that is based on one of the NDT techniques i.e., machine vision. The most evident components of the specified system are the image acquisition system and image processing system. The image acquisition system constitutes an assembly of hardware i.e., cameras and sources of light. Image preprocessing, image analysis using ML and DL models are all included in the image processing module to identify defects in the recorded image data (Bai et al., 2025). The results are evaluated using various parameters as discussed in Section 4.3.

**Figure 1:**
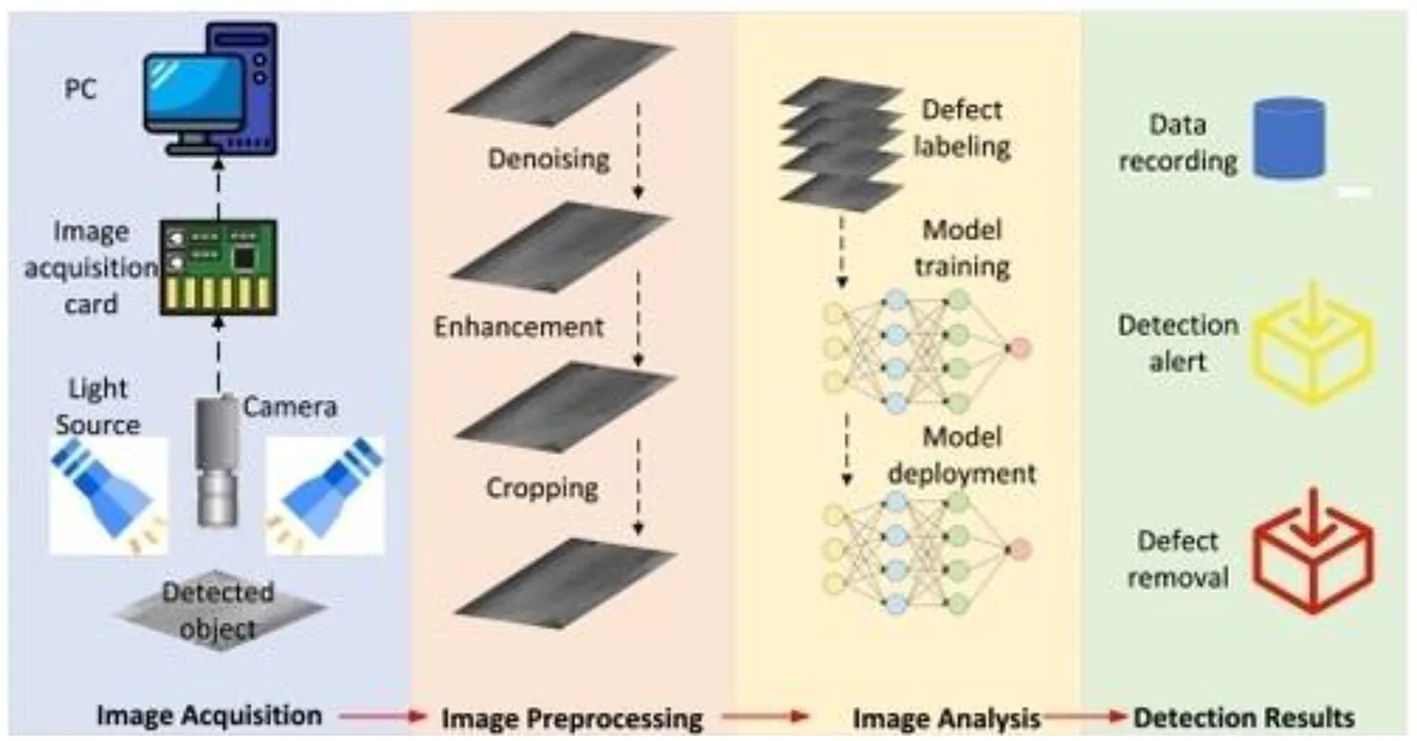
Process of defect detection using machine learning and NDT (Bai et al., 2025). Licensed under Copyright@2025; Bai et al.; Requested author for permission to reproduce. Source: https://doi.org/10.1145/3730576 (Bai et al., 2025).

### 1.6 Review aim and research questions

The aim of the study is to critically consolidate and synthesize the rapidly expanding body of literature on data-driven algorithmic advances in defect detection of additively manufactured materials using NDT, in order to provide an organized point of reference for researchers and practitioners to understand the cutting edge phenomenon, identify methodological gaps, and select appropriate ML-NDT combination for specifics AM applications. To achieve this aim, this review addresses the research questions as follows:

RQ1) What are the different NDT techniques used in defect detection of AM materials? RQ2) What are the various machine learning algorithms which can be integrated with NDT techniques? RQ3) What are the different challenges in defect detection of AM materials?

The outline of the proposed review is discussed here. The review methodology of this proposed study along with classification criterion for literature review is described in Section 2. Section 3 summarizes various studies on defect detection methods used on various AM materials whereas methodology employed by various studies and a comparison of different NDT techniques and machine learning algorithms based on performance metrics, advantages, and limitations is discussed in Sections 4 and 5, respectively. Section 6 describes the overall discussion on NDT techniques, ML/DL algorithms and challenges in defect detection while the most mature ML-NDT combinations, the main limitations of current research and the most important future prospects in research are concluded in Section 7.

## 2 Review methodology

This review followed a structured literature identification and screening process informed by systematic review conventions, adapted for a focused technical domain. It applies four preliminary steps: search, screening, synthesis and analysis. Each step is described in subsequent subsections.

### 2.1 Search strategy

The scope of this study is defined to form the research questions mentioned in the previous section clearly and answerable. In view of this, a comprehensive literature survey is conducted across Google Scholar, IEEE Xplore, Science Direct, ACM digital library for the publications in the years from 2012 to 2026. The search strategy is designed around four components: Additive Manufacturing (AM), monitoring stage, NDT techniques, and data-driven AI algorithms. The search keywords used are “additive manufacturing,” “3D printing,” “non-destructive testing,” “machine learning,” “deep learning,” “artificial intelligence,” and “data-driven.”

### 2.2 Inclusion and exclusion criteria

To make this study more focused, an eligibility criterion is established before screening as mentioned in Table 1.

**Table 1:** Inclusion and exclusion criteria.

Inclusion criteria	Exclusion criteria
Focus on AM process	Non-AM processes
Use of NDT or *in-situ* monitoring modality	Visual or manual inspection
ML/DL algorithms for defect detection	Mechanical property studies of materials, parameter optimization studies
Peer-reviewed Journal Articles and Reviews Fully indexed conference proceeding papers, theses	Notes, Book Chapters, Patents, Editorial, and non-English papers

### 2.3 Screening results

After the application of search string and the above criteria, the screening of title and abstract and full-text eligibility evaluation are carried out. A total of 50 studies comprising 48 peer-reviewed journal or conference papers and 2 theses published in English language are featured for full inclusion.

### 2.4 Synthesis

After receiving the final pool of literature, the research/review articles, conference papers and theses are synthesized based on the classification criteria described in Table 2.

**Table 2:** Classification criteria for literature review.

Primary dimension	Classification sub-criteria	Typical research targets / keywords
1. Manufacturing process	Process type (ASTM categories)	Powder Bed Fusion (PBF), Directed Energy Deposition (DED), Material Extrusion (FDM/FFF), Binder Jetting, Selective Laser Melting (SLM), Wire and Arc Manufacturing (WAAM).
	Feedstock material	Metals (SS316, Inconel718, Ti-6Al-4V, Maraging Steel C300), Polymers (Nylon, PLA, PA12), Composites (CFRP), Ceramics.
2. NDT modality	Sensing technology	Thermography (IR), Ultrasonic Testing (UT), X-ray Computed Tomography (XCT), Acoustic Emission (AE), Computer Vision.
3. Machine learning task	Learning paradigm	Supervised (Classification/Regression), Unsupervised (Clustering/Anomalies), Semi-supervised, Physics-informed Machine Learning (PIML).
	ML architecture	Random Forest (RF), Support Vector Machine (SVM), Deep Learning (CNN, U-Net), Recurrent Neural Network (RNN)/Long Short-Term Memory (LSTM), You Look Only Once (YOLOv), Regression Algorithm.
4. Monitoring stage	Implementation timing	***In-situ*****:** Real-time melt pool or powder bed monitoring. ***Ex-situ*****:** Post-build certification and final inspection.
5. Defect characteristics	Defect type	Porosity, Lack of Fusion (LoF), Delamination, Cracks, Balling, Stringing, Voids, Fiber Direction Misalignment, Underfill, Overfill, Low Layer times.
6. Data strategy	Data source	Experimental Data, Synthetic Data (Finite Element Aanalysis/Simulation), Hybrid/Augmented Data.
7. Performance evaluation	Optimization focus	Accuracy, Precision, Recall, F1-score, Mean Absolute Error (MAE), Mean Square Error (MSE), Root Mean Squared Error (RMSE).

### 2.5 Analysis

The content analysis based on the methodologies used by the studies for dataset preparation using NDT techniques and implementation of ML/DL algorithms is described in the Section 4.

## 3 Literature review

The monitoring stages for defect identification in AM materials can be *in-situ, ex-situ*, or in- service. Considering *in-situ* monitoring, Pandiyan et al. (2021) and Chen et al. (2023) used acoustic emission integrated with two designs of generative CNN based on the General Adversarial Network (GAN) and Variational Auto-Encoder (VAE) for online prediction of balling, LoF, keyhole pores defects in laser powder bed fusion (L-PBF) and CNNs for cracks in laser directed energy deposition (L-DED) processes, respectively. In another study, the printing defects including spatter and delamination, were detected with 96.80% accuracy by combining thermographic off-axis imagery as a dataset with neural network designs based on deep learning (Baumgartl et al., 2020). Successively, Ho et al. (2021) investigated the use of deep learning (DL) algorithms to predict porosity in metal AM processes by analysing thermal images whereas Klamert (2025) applied deep learning for defect detection and real-time monitoring of the Selective Laser Sintering (SLS) AM process utilizing thermal infrared imaging and laser profilometry. For predicting porosity in metal-based L-PBF additive manufacturing, infrared thermography or short-wave infrared thermography (SWIT) were integrated with deep learning algorithms like CNNs (Oster et al., 2024a,b). Similarly, Chen et al. (2019) presented a passive infrared thermography method focusing on real-time detection of shape defects in molten layers of WAAM manufactured carbon steel component. The AlexNet model was used to classify defects and ensure quality control by adjusting processing parameters based on detection results.

While considering *ex-situ* monitoring, Lin et al. (2023) employed a new multiscale convolutional fuzzy neural network (MCFNN) for quality assurance in metallic components produced using SLM process. Ultrasound images were utilized to assess internal defects, achieving high accuracy and efficiency compared to existing models. Another study investigated the use of thermal tomography (TT) for non-destructive evaluation of additive manufactured (AM) stainless steel 316L structures, focusing on detecting internal porosity defects using sparse coding (SC) and K-Singular Value Decomposition (K-SVD) algorithm (Zhang et al., 2020a) while Chen et al. (2021) employed a micro-CT scan and deep learning architectures including RNN and CNN, for an additively manufactured fiber- reinforced composite material to detect defects such as voids and misaligned fiber orientations. Likewise, deep learning methods such as YOLOv5 and U-net were initially developed by Acharya et al. (2022) to detect and segment porosity defects in XCT images of AM parts while Zhu et al. (2020) employed an unsupervised learning algorithm i.e., PCA and clustering analysis for characterizing defect evolution in binder jetting AM process integrating fast X-ray CT and computer vision methods.

AI-based computer vision was used as a novel method for detecting stringing defects of a fused filament fabrication (FFF) printed object along with a Deep Convolutional Neural Network (DCNN) (Paraskevoudis et al., 2020). Moreover, contactless (remote) mechanical testing and fracture prediction in 3D construction materials utilizing thermal imaging data were demonstrated by Boiko et al. (2022). To forecast fractures, a fast convolutional neural network (FCNN) was created.

Additionally, Szymanik et al. (2021) presented a novel approach based on the fusion of active infrared thermography (IRT) and DCNN to detect surface-breaking holes with different sizes and depths. In succession, Szymanik (2022) outlined a study using active infrared thermography. In this study, Polyethylene Terephthalate Glycol (PETG) sample was assessed using halogen lamps as a source of excitation for assessing the structural characteristics of 3D-printed materials along with a neural network, specifically LSTM technique. Furthermore, using temperature data from thermal images, a method based on step-heating thermography was introduced to measure the length dimension of small AM defects (Rodríguez-Martín et al., 2022). Unsupervised learning algorithms such as Spatial-Temporal Blind Source Separation (STBSS), Spatial-Temporal Sparse Dictionary Learning (STSDL), Spatial temporal denoised thermal source separation (STDTSS), and Spatial-temporal separation-based clustering segmentation (STSCS) were employed with pulsed thermography for detecting porosity in metal-based L-PBF manufactured AM components (Heifetz et al., 2020; Zhang et al., 2021, 2024).

Meanwhile, Kapusuzoglu and Mahadevan (2020) applied physics-informed and hybrid ML to FFF process defect prediction while Liu et al. (2021) presented a physics-informed ML (PIML) model specifically to predict porosity in L-PBF AM. Recently, Farrag et al. (2025) reviewed PIML applications across metal AM while Guo et al. (2022) framed this as a shift toward a physics-informed, data-driven paradigm for metal AM components more broadly. Leng et al. (2026) offered a thorough analysis of physics-informed ML across intelligent manufacturing, setting AM-specific predictive maintenance applications within this wider methodological trend. Table 3 describes the NDT methods and machine learning algorithms categorized based on monitoring stage of defect detection.

**Table 3:** NDT methods and machine learning algorithms categorized based on monitoring stage of defect detection.

References	Stage	NDT technique	Material	Process	ML architecture	Defect
Pandiyan et al. (2021)	*In-situ*	Acoustic emission	Inconel 718	L-PBF	Generative CNN: VAE and GANomaly	Balling, LoF, keyhole pores
Chen et al. (2023)	*In-situ*	Acoustic signals	Maraging steel C300	L-DED	CNN	Crack and Keyhole
Baumgartl et al. (2020)	*In-situ*	Thermal imaging	1.2344 (H13)	L-PBF	CNN	Delamination metal spatters
Ho et al. (2021)	*In-situ*	Thermal imaging	Ti-6Al-4V	PBF	Res-RCNN	Porosity
Klamert (2025)	*In-situ*	Thermal imaging and laser profilometry	PA12 (PA 2200)	L-PBF/ SLS	CNN	Curling
Oster et al. (2024a)	*In-situ*	IR thermography	AISI 316L Stainless Steel	L-PBF	CNN	Porosity
Oster et al. (2024b)	*In-situ*	Shortwave infrared thermography	Nickel-based superalloy	PBF-LB/M	Random Forest	Porosity
Chen et al. (2019)	*In-situ*	Passive IR thermography	Carbon steel	WAAM	CNN-AlexNet	Shape defects
Lin et al. (2023)	*Ex-situ*	Ultrasound	Inconel 718	SLM	Fuzzy Neural Network	Quality assessment
Zhang et al. (2020a)	*Ex-situ*	Thermal tomography	Stainless steel 316L	PBF	Sparse Coding (SC) K-SVD	Porosity
Chen et al. (2021)	*Ex-situ*	Micro-CT	ABSGF10 Glass Fiber-Reinforced	FFF	RNN CNN	Fiber direction misalignment and voids
Acharya et al. (2022)	*Ex-situ*	XCT	cobalt chrome	L-PBF	YOLOv5	Porosity
Zhu et al. (2020)	*Ex-situ*	XCT, Computer vision	Copper	Binder Jetting	PCA and Clustering Analysis	Porosity
Paraskevoudis et al. (2020)	*Ex-situ*	Computer vision	Not specified	FFF	DCNN	Stringing
Boiko et al. (2022)	*Ex-situ*	Thermal imaging	Not specified	FDM	FCNN/LSTM	Fracture prediction
Szymanik et al. (2021)	*Ex-situ*	Active IR thermography	ABS plastic	Parallel Lines Pattern	3D-CNN DID-CNN DED-CNN	Surface- breaking holes
Szymanik (2022)	*Ex-situ*	Active IR thermography	PETG	FFF	LSTM	Holes
Rodríguez-Martín et al. (2022)	*Ex-situ*	Step-Heating Thermography	Nylon and PLA	Not specified	SVM, Linear Regression, Gaussian regression	Internal defect size measurement
Heifetz et al. (2020)	*Ex-situ*	Pulsed thermography	Stainless steel 316L and Inconel 718	L-PBF	STBSS Algorithm STSDL Algorithm	Porosity
Zhang et al. (2021)	*Ex-situ*	Pulsed thermography	Stainless steel 316L	L-PBF	STDTSS Algorithm	Porosity
Zhang et al. (2024)	*Ex-situ*	Pulsed infrared thermographic microscopy	(SS316) and Inconel 718(IN718)	L-PBF	STSCS algorithm	Porosity
Liu et al. (2021)	*Ex-situ*	Computed tomography	Inconel 718	PBF	PIML	Porosity

## 4 Methodology

The research methodology and techniques utilized by machine learning algorithms for AM material defect detection are outlined in this section. This discusses the simulations and experimental setups of different NDT techniques coupled with machine learning models.

### 4.1 Dataset preparation using NDT techniques

Machine learning models employ various data sources, including thermal images and sensor readings. For instance, throughout the L-PBF process, a number of sensors were used for real-time defect monitoring by Everton et al. (2016). The airborne acoustic emission (AE) of the L-PBF process zone was measured using an airborne resonant acoustic sensor while acoustic signals were recorded using an Advantech Data Acquisition (DAQ) card. These signals were analyzed for anomaly identification in Inconel 718, a nickel-based superalloy (Pandiyan et al., 2021) whereas Chen et al. (2023) employed an *in-situ* robotic acoustic monitoring system which was used for generating dataset of L-DED processed metal components.

Furthermore, Zhang et al. (2020a) utilized a low-cost infrared camera having a spatial resolution of 640 × 512 pixels and imaging frame rate of 30 Hz, with a resolution of approximately 140 μm per pixel to enhance imaging quality and addressed potential material flaws that could affect performance in nuclear applications.

A 20-MHz single-element ultrasound transducer, a pulser, X-Y-Z motor stages, a motor controller and an 8-bit analog-to-digital converter were the primary components of the high-frequency ultrasound imaging system, which was created in another experimental study for quality assessment of metallic components manufactured by SLM process (Lin et al., 2023).

Considering another NDT technique, XCT, Chen et al. (2021) employed a printed sample that was scanned using a μCT scanner. A total of 22,806 μCT images of 100 × 100 pixels each were used to generate dataset. Similarly, Acharya et al. (2022) segmented and identified porosity defects in L-PBF manufactured cobalt chrome components using XCT images whereas Zhu et al. (2020) used a micron-scale XCT platform to scan the 3D defect morphology for copper sample.

Using a Prusa i3 MK3S 3D printer, Paraskevoudis et al. (2020) illustrated AI-based computer vision to capture images of printed products with defects. As a training dataset, 500 images exhibiting a stringing phenomenon were collected. In another study, two thermal imaging devices were put up to acquire *in-situ* thermal data. Labeled 1,557 images with 72 containing porosities were captured by an infrared camera and HAZ images by the pyrometer (Ho et al., 2021).

To create a dataset of artificially created curling defects, a FLIR T420 thermal infrared camera was used to record the temperature distribution at the powder bed's surface by Klamert (2025) whereas Boiko et al. (2022) presented a demonstration of contactless (remote) mechanical testing and fracture prediction in 3D construction plastic materials using thermal data.

Baumgartl et al. (2020) also utilized the middle infrared range of the infrared camera for capturing images. A convolutional neural network (CNN) was applied and evaluated using a dataset of 4,314 RGB color images. Eighteen short clips of video displaying splatters, delamination, or flawless images were taken from the original video. Furthermore, a dataset generated for thermographic in-process monitoring of the production of an AISI 316L stainless steel test specimen manufactured by L-PBF process was utilized by Oster et al. (2024a). The size of the dataset used ranged from 4,972 samples to 836,426 samples which depends on the chosen cluster edge length.

The study carried out by Oster et al. (2024b) as well used shortwave Infrared thermography (SWIR) during the experiments on a metal-based PBF with laser beam (PBF-LB/M) printer for an off-axis *in-situ* monitoring to generate the dataset for porosity prediction in metallic components. Moreover, Szymanik et al. (2021) also used active thermography excited by a halogen lamp for 3D-printed sample having a set of manufactured defects. Vectors of 361 thermal images were registered for each defective location and obtained throughout the test period to create the source data set. In another study, the numerical model developed in COMSOL was utilized to identify holes. In the experimental setup, 360 images of temperature distribution for model training were generated using halogen lamp used as a heat source. The final database included 2,56,500 features pertaining to defects and 2,56,500 features pertaining to background (Szymanik, 2022). The tests performed by Chen et al. (2019) were based on a Yaskawa robot and a LORCH welder for WAAM manufactured carbon steel component to detect shape defects. Furthermore, Rodríguez-Martín et al. (2022) generated large data sets using the finite element method for training ML algorithms whereas Heifetz et al. (2020) and Zhang et al. (2021) used the experimental setup of a mid-wave infrared (MWIR) imaging camera, the FLIR X8501sc, which captures time-resolved images. As a result, the obtained thermal-imaging data cube was made up of several two- dimensional images of the surface temperature captured at various intervals. Zhang et al. (2024) as well used a low cost compact FLIR A65 camera which had dimensions of 295L × 200W × 105H mm to capture images.

### 4.2 Machine learning models used

There are three kinds of machine learning algorithms: supervised, unsupervised, and semi-supervised (Saberironaghi et al., 2023). In the supervised approach, CNNs have evolved as the leading solution for resolving several challenging computer vision issues in recent years. Considering the complex structure of the human brain's visual cortex, CNNs have demonstrated impressive performance in a variety of extremely challenging application scenarios (Baumgartl et al., 2020). Specifically, neural networks are made to identify 3D printing defects by examining video taken during the printing process (Paraskevoudis et al., 2020). Figure 2 depicts a standard CNN architecture used in one of the studies to classify the defects.

**Figure 2:**
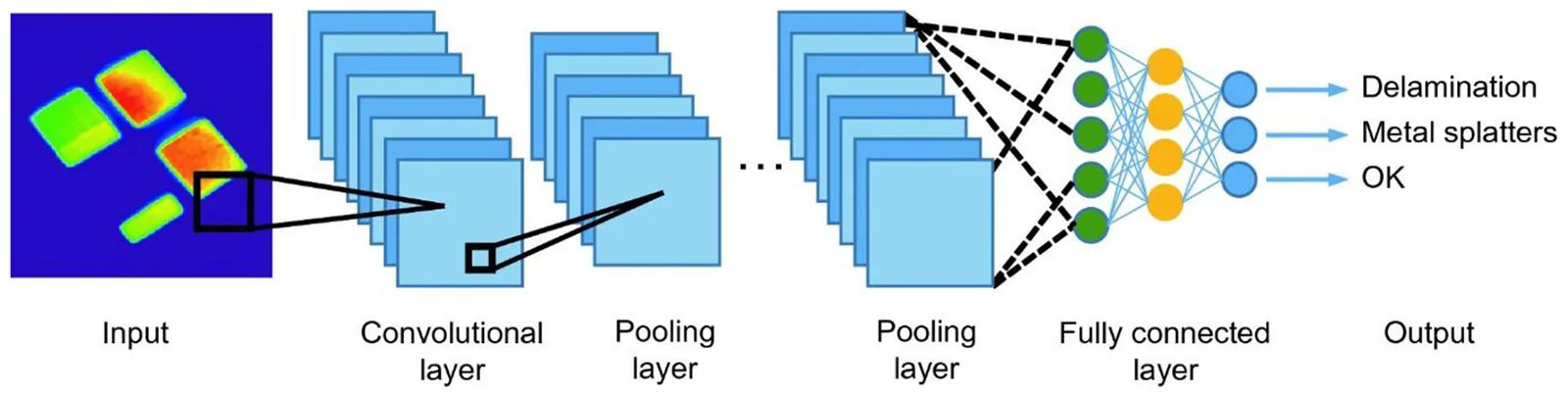
An illustration of a standard CNN architecture with convolutional layer blocks, pooling layers, and fully connected layers (Baumgartl et al., 2020). Licenced under CC By 4.0: http://creativecommons.org/licenses/by/4.0/. Source: https://link.springer.com/article/10.1007/s40964-019-00108-3 (Baumgartl et al., 2020)

With an emphasis on acoustic signal denoising and feature extraction, Chen et al. (2023) provided an acoustic-based *in-situ* defect detection technique that uses a CNN for real-time defect prediction during the L-DED process. The CNN model with the highest overall accuracy of 89% in defect prediction was trained on the denoised dataset. With the CNN model, keyhole pore prediction accuracy reached 93%. The AUC-ROC score for the CNN model was reported as 98% (Chen et al., 2023). Another study also involved training of CNNs to predict defects with high accuracy, demonstrating the potential for automated process monitoring (Klamert, 2025). Remarkably, Oster et al. (2024a) investigated CNN-based machine learning method for predicting keyhole development. In a different study, Oster et al. (2024b) processed raw images using a combination of dense regression, LSTM and convolutional layers directly. It was concluded that this method eliminated the need for manual feature extraction, which would have increased computing complexity.

In order to provide an online defect detection in a manufacturing process, Chen et al. (2019) used passive IRT along with CNN. This method allowed the closed-loop feedback on the AM process by adjusting the subsequent layer's processing parameters based on data detected from the current layer.

Baumgartl et al. (2020) employed machine learning (ML) methods for error recognition in the L-PBF process, using thermographic imaging data as the primary data source. A combination of deep learning-based neural network architectures was used for delamination defect detection with 96.80% accuracy. The architecture was made to be compact and effective allowing real-time operation even on less powerful hardware. The network was trained using the TensorFlow 1.8 backend and the Keras 2.1.5 package. In order to characterize anomalous regimes, Pandiyan et al. (2021) illustrated the use of generative CNN architectures, variational autoencoder (VAE) architectures and GANomaly whereas Chen et al. (2021) demonstrated ANN architectures in two varieties: RNNs and CNNs which were used to construct models with Python and TensorFlow.

Furthermore, Ho et al. (2021) explored deep learning techniques, specifically CNN utilizing transfer learning along with Residual-Recurrent Convolutional Neural Networks (Res-RCNN), to predict porosity in real-time for AM processes. The methodology included analysis of thermal images of the melt pool to interpret temperature variations during the layer-by-layer fabrication of metal parts.

Lin et al. (2023) examined Inconel 718 components produced by selective laser melting (SLM) under different process conditions using a unique multiscale convolutional fuzzy neural network (MCFNN) and ultrasound images.

Szymanik et al. (2021) presented a novel approach based on the fusion of active infrared thermography (IRT) and deep convolutional neural networks (DCNN) to detect surface breaking holes with different sizes and depths. A deep convolutional neural network-based multi-label classifier and a specific algorithm for signal analysis were used in conjunction with the suggested novel IRT technique. Moreover, neural networks were trained on annotated images generated by AI-based computer vision to identify stringing defects, utilizing a dataset created from a “Stringing test” object (Paraskevoudis et al., 2020).

Additionally, Szymanik (2022) employed neural networks, specifically LSTMs and achieved accuracy of 91.48% for 100% infill density and 87.43% for 30% infill density whereas Boiko et al. (2022) achieved Mean Absolute Error (MAE) of 0.37 and Root Mean Squared Error (RMSE) of: 0.48 using FCNN integrated with LSTM.

Furthermore, Acharya et al. (2022) tackled the issue of defect detection in XCT images using a single-stage object detection technique (YOLOv5). Three distinct YOLOv5 variations were used and their results were contrasted. U-Net was used to segment defects in slices of XCT. In another study, the authors reviewed a segmentation methodology using U-Net for defect detection across various AM processes, enhancing data diversity and reducing manual labeling efforts (Deshpande et al., 2024). ML algorithm was used to reduce thermographic noise while identifying metallic component defects (Zhang et al., 2020b, 2021) while, Rodríguez-Martín et al. (2022) predicted the length of the defects using regression learners in various configurations. Bayesian inference was used to optimize the predictive methods that were obtained.

Moreover, Heifetz et al. (2020) used unsupervised machine learning algorithms, including the novel STBSS and STSDL techniques, to study the extraction of low SNR (signal-to- noise ratio) features that correspond to material flaws in thermal images, such as porosity of different sizes. Principal component analysis (PCA) was employed to categorize detected pores into morphological groups, enhancing the understanding of defect characteristics (Zhu et al., 2020) while, Zhang et al. (2021) employed STDTSS algorithm to reduce thermographic noise while identifying metallic component defects. Similarly, an unsupervised learning (UL) algorithm, STSCS was utilized, which comprises of independent component analysis (ICA), principal component analysis (PCA), and wavelet transformation and segmentation to enhance defect visibility (Zhang et al., 2024).

Lastly, rather than using machine setting parameters directly for prediction of porosity levels of printed products, a physics-informed data-driven model (PIM) first translated machine setting parameters into physical effects such as laser energy density and laser radiation pressure. PIM performed well with a 10%−26% prediction error after applying six learning approaches (Liu et al., 2021).

### 4.3 Performance evaluation metrics

The evaluation of various machine learning algorithms, including CNNs and other algorithms can be carried out to determine their effectiveness in real-time as well as post-production defect detection in 3D printed components. The assessment of these models provides insights about their performance and suitability for detecting defects in various AM materials. Performance evaluation metrics such as F1-score, accuracy, precision and recall (Equations 1–4) are used to assess how well machine learning algorithms detected defects through a confusion matrix as described in Table 4.

**Table 4:** Confusion matrix.

Confusion matrix	Actual	
Predicted	True Positive (TP)	False Positive (FP)
	False Negative (FN)	True Negative (TN)

The true positive is regarded as a defect whereas the false positive is regarded as identifying the background or part of the image slice as a defect. Precision quantifies the model's accuracy in making the correct detections whereas recall indicates how effectively the model detects all defects in the image. The range of values for precision and recall is between 0 and 1. However, the Intersection of Union (IoU) threshold has an impact on all these indicators (Equation 5). The performance of object detection models is assessed using statistics known as Mean Average Precision (mAP) (Equation 6). It quantifies how accurately a model can identify and locate objects within an image by combining precision and recall. mAP@50 is calculated using an Intersection over Union (IoU) threshold of 0.5, while mAP@.5:.95 is computed across several IoU thresholds, usually from 0.5 to 0.95 with a step of 0.05, giving a more comprehensive evaluation. The following expressions show the mathematical relation of described metrics (Acharya et al., 2022).


$$\begin{array}{l} \text{Precision}=\frac{\text{TP}}{\text{TP }+\text{FP}} \end{array}$$ (1)

$$\begin{array}{l} \text{Accuracy}=\frac{\text{TP }+\text{ TN}}{\text{TP}+\text{FP}+\text{TN}+\text{FN}} \end{array}$$ (2)

$$\begin{array}{l} \text{Recall}=\frac{\text{TP}}{\text{ TP}+\text{FN}} \end{array}$$ (3)

$$\begin{array}{l} \text{F}1-\text{Score}=\;\frac{2\;*Precision*Recall\;}{\text{Precision }+\text{ Recall}} \end{array}$$ (4)

$$\begin{array}{l} IoU=\frac{TP}{TP+FP+FN} \end{array}$$ (5)

$$\begin{array}{l} mAP=\frac{1}{N}\overset{N}{\underset{i}{\sum }}AP_{i} \end{array}$$ (6)

*where, N: No. of classes* *AP*_*i*_*: Average precision of class i*

In regression analysis, the performance of the model is evaluated through the Mean Absolute Error (MAE), Mean Squared Error (MSE), Root Mean Squared Error (RMSE) and R-Square or Coefficient of Determination (R^2^) metrics (Equations 7–10) (Szymanik, 2022).


$$\begin{array}{l} \text{MAE}=\;\frac{1}{N}\overset{N}{\underset{I=1}{\sum }}\left(y_{i}-{ŷ}_{i}\right) \end{array}$$ (7)

$$\begin{array}{l} \text{MSE}=\;\frac{1}{N}\overset{N}{\underset{I=1}{\sum }}\left(y_{i}-{ŷ}_{i}\right)2 \end{array}$$ (8)

$$\begin{array}{l} \text{RMSE}=\;\sqrt{MSE}\text{=}\sqrt{\frac{1}{N}\overset{N}{\underset{I=1}{\sum }}\left(y_{i}-{ŷ}_{i}\right)2} \end{array}$$ (9)

$$R2=1-\frac{\sum \text{​}\left(y_{i}-{\hat{y}}_{i}\right)2}{\sum \text{​}\left(y_{i}-\;y_{i}\;\right)2}$$ (10)

Where, *y*_*i*_ :Actual value *y*_*i*_: Predicted value

${\bar{y}}_{i}$ *: Mean value N : No. of data points*

## 5 Comparative analysis

This section summarizes the performance evaluation of ML algorithms and advantages, limitations and suitability of each ML-NDT combination reported in the literature.

### 5.1 Performance evaluation of ML algorithms

Table 5 summarizes reported performance metrics across principal ML model families identified in the reviewed literature. Because studies differ substantially in material, AM process, dataset size, defect type, NDT technique, and validation protocol, the reported accuracy ranges should be interpreted as indicative rather than directly comparable benchmark scores; nonetheless clear patterns are emerged. CNN-based deep learning models consistently report the highest classification accuracies (~90%−99%) among supervised approaches, reflecting their strength in extracting spatial features from thermographic, optical and CT image data (Baumgartl et al., 2020; Chen et al., 2021; Acharya et al., 2022). Hybrid architectures like the multiscale convolutional fuzzy neural network achieve comparable accuracy (~90%−96%) while offering improved robustness to ultrasonic signal ambiguity (Lin et al., 2023). Unsupervised and semi-supervised approaches are harder to benchmark quantitively but can be used for early stage process monitoring where large labeled datasets do not yet exist (Pandiyan et al., 2021; Heifetz et al., 2020). Physics-informed ML approaches, while less uniformly benchmarked using a single accuracy metric are consistently reported to improve generalization and extrapolation performance relative to purely data-driven models (Farrag et al., 2025; Guo et al., 2022; Liu et al., 2021; Kapusuzoglu and Mahadevan, 2020).

**Table 5:** Comparison of machine learning algorithms used for AM materials.

References	NDT technique	Methodology	Performance/results
Chen et al. (2023)	Acoustic signals	CNN	Accuracy: 89%
Pandiyan et al. (2021)	Acoustic emission	Generative CNN architectures: VAE and GANomaly	Accuracy: VAE: 96%, GANomaly: 97%
Klamert (2025)	Thermal imaging & laser profilometry	CNN	Accuracy: 99%
Ho et al. (2021)	Thermal imaging	Res-RCNN	Accuracy: 99.49%
Oster et al. (2024a)	IR Thermography	CNN	Accuracy: 96 %, F1-Score : 86%
Oster et al. (2024b)	Shortwave infrared thermography	Random Forest	The RF model: RMSE = 0.537% R^2^ score = 0.913 LR algorithm: R^2^ score = 0.849
Baumgartl et al. (2020)	Thermography	CNN	Accuracy: 96.80%.
Chen et al. (2019)	Passive IR thermography	CNN-AlexNet	Accuracy: 91%
Lin et al. (2023)	Ultrasound testing	Fuzzy Neural Network	Accuracy: 91.44%; Precision: 92.74% Recall: 91.44%; F1-score: 91.35%
Chen et al. (2021)	Micro-computed tomography	RNN CNN	RNN: Training MSE: 0.0074 Validation MSE: 0.0128, Test MSE: 0.0154 CNN: Training MSE: 0.01494 ValidationMSE: 0.00536, Test MSE: 0.00036
Acharya et al. (2022)	X-ray Computed Tomography (XCT)	YOLOv5 U-net	YOLOv5s: mAP:88.45% at IoU 0.5 YOLOv5m: Average precision: 0.95 at IoU0.5 to 0.95 YOLOv5s: Recall: 87.65% U-Net accurately segments defects from XCT slices
Paraskevoudis et al. (2020)	AI-based computer vision	DCNN	Average Precision = 0.4, IoU threshold = 0.6 Precision = 0.75, Recall = 0.92
Boiko et al. (2022)	Thermal imaging	FCNN//LSTM	MAE: 0.37 RMSE: 0.48
Zhang et al. (2024)	Pulsed infrared thermographic microscopy	STSCS algorithm	Precision: 1, Recall: 0.5, F-score: 0.25
Rodríguez-Martín et al. (2022)	Step-heating thermography	SVM Linear Regression Gaussian Regression	The defect size varies between 13% and 37% for Polylactic Acid (PLA) and between 13% and 36% for Nylon
Szymanik (2022)	Active IR thermography	Neural networks (LSTM)	Accuracy: For 100% infill density: 91.48% For 30% infill density: 87.43%
Szymanik et al. (2021)	Active IR thermography	3D-CNN DID-CNN DED-CNN	Accuracy: 3D-CNN: 99.20% DID-CNN: 98.97% DED-CNN: 99.41%
Heifetz et al. (2020)	Pulsed thermography	STBSS Algorithm STSDL Algorithm	Smaller defects have a slightly higher F-score for STBSS, whereas larger defects have a slightly higher F-score for STSDL.
Liu et al. (2021)	Computed tomography	PIML	Prediction error 10%−26%

The reported performance values originate from different datasets AM processes, NDT techniques, defect types, and validation protocols and therefore should not be considered as directly comparable benchmarks.

Despite these concerns, some tentative, qualitative patterns are visible. CNN-based architectures including plain CNN, CNN-AlexNet, 3D/DID/DED-CNN variants, Res-RCNN, DCNN dominate the methodology and are applied across almost every NDT modality indicating that convolutional approaches are the current default choice for image-like AM inspection data i.e., thermal frames, CT slices, camera images (Chen et al., 2019, 2021; Baumgartl et al., 2020; Szymanik et al., 2021; Ho et al., 2021; Lin et al., 2023; Klamert, 2025). Recurrent/sequence models (RNN, LSTM, FCNN/LSTM) tend to appear where the signal is inherently time-series in nature, such as acoustic signals or thermal sequences (Chen et al., 2021; Szymanik, 2022; Boiko et al., 2022). Generative anomaly detection architectures including VAE and GANomaly appear specifically where labeled defect examples are scarce and consistent with their use in a semi-supervised acoustic emission setting (Pandiyan et al., 2021) whereas classical regression ML algorithms including Random Forest, SVM, Gaussian Regression, Linear Regression, are reserved for defect-sizing or property prediction tasks rather than binary defect detection (Rodríguez-Martín et al., 2022; Oster et al., 2024b). Finally, it is observed that *in-situ* monitoring more often report a single accuracy figure aimed at fast real-time decision making, while *ex-situ* monitoring more often report richer metrics including precision, recall, sizing error, reflecting the different purpose of post-build characterization vs. in-process monitoring.

### 5.2 Advantages and limitations of ML-NDT combinations

This section summarizes the principal ML-NDT combinations reported in the literature and discusses the advantages, limitations, and suitability of each ML-NDT combination.

#### 5.2.1 Infrared/thermographic imaging combined with ML

Infrared thermography which is used as an *in-situ* melt pool monitoring technique and as a post-process active/pulsed thermography inspection is the most extensively reported ML-NDT pairing in the reviewed literature.

##### 5.2.1.1 *In-situ* thermographic monitoring

It is mainly used for metal L-PBF/DED and WAAM processes where early detection of porosity, cracking or lack-of-fusion is valuable. Deep learning models trained on *in-situ* thermographic image streams from L-PBF and DED are used to report anomalous melt-pool signatures and predict porosity as the part being built (Baumgartl et al., 2020; Ho et al., 2021; Oster et al., 2024a,b). Passive infrared monitoring is applied to wire-arc additive manufacturing (WAAM) for online defect detection (Chen et al., 2019).

##### Advantages

Thermographic imaging integrated with ML enables real-time feedback during the AM process, supports closed loop process control rather than only post-build inspection and sensitive to thermal signatures associated with keyhole, lack-of-fusion, and porosity formation before the part is complete (Chen et al., 2023; Ho et al., 2021; Oster et al., 2024a). Compact IR camera and pulsed thermography setups shows good agreement with ML classification of subsurface defects at comparatively low hardware cost (Zhang et al., 2020a,b).

##### Limitations

Signal quality in thermal imaging is highly sensitive to surface emissivity, powder spatter, which can be confused with genuine defect signatures (Szymanik et al., 2021; Szymanik, 2022) and thermal diffusion blurs small or deep features as well. Most reported models are trained on relatively small datasets, limiting generalization across machines and materials (Klamert, 2025; Oster et al., 2024b). Moreover, comparative studies show performance varies remarkably between ML techniques i.e. classical ML vs. deep networks for the same thermal dataset, so results are not always transferable.

##### 5.2.1.2 Post-process active/pulsed thermography

Post-process active or pulsed thermography which is often combined with simulation informed or numerically trained neural networks is used to size and localize internal defects in printed polymers and metals (Rodríguez-Martín et al., 2022; Szymanik, 2022; Heifetz et al., 2020; Zhang et al., 2024) and is well suited as a fast, low cost screening step for post-build quality assurance, particularly for polymer AM and for complex or curved metal geometries (Szymanik, 2022; Montinaro et al., 2018).

##### Advantages

This method can be trained on numerically simulated thermal responses, reducing dependence on large experimental defect libraries (Szymanik, 2022). Step-heating and pulsed approaches allow estimation of defect depth and size, not just presence or absence (Zhang et al., 2024).

#### Limitations

As this method requires an external heating/excitation source and controlled ambient conditions, it is inherently an offline, post-build inspection step (Rodríguez-Martín et al., 2022; Cerniglia and Montinaro, 2018). Also, the accuracy of ML-based sizing degrades for defects close to the surface-to-surface boundary or with irregular geometry.

#### 5.2.2 Computer vision /optical surface imaging combined with ML

Camera based visual /optical monitoring which is analyzed with classical computer vision or deep neural networks targets surface and near surface defects including cracks, voids, and delamination, typically in polymer material-extrusion and ceramic printing as well as fiber-reinforced composite AM (Paraskevoudis et al., 2020; Zhou et al., 2024; Chen et al., 2021; Borish et al., 2020; Okarma and Fastowicz, 2019).

#### Advantages

This is low-cost camera hardware that can be fitted onto existing printers. High acquisition rate of this method supports real-time, remote or layer-by-layer defect detection during printing, including stringing and warping defects (Paraskevoudis et al., 2020; Borish et al., 2020).

#### Limitations

This is fundamentally a surface inspection technique, it cannot detect internal porosity, subsurface cracking or lack-of-fusion (AbouelNour and Gupta, 2022; Okarma and Fastowicz, 2019). Model performance reported in the literature is often demonstrated on a single material/geometry class raising questions about generalizability (Chen et al., 2021).

#### 5.2.3 Optical and confocal microscopy combined with ML

Confocal and scanning microscopy is used to characterize AM surface topography and to quantify porosity. These techniques are typically used for high-resolution metrology and ground truth characterization and provides valuable labeled data for training vision and thermography based ML models (Grimm et al., 2015; Wang et al., 2018).

#### 5.2.4 X-ray computed tomography combined with ML

X-ray Computed tomography (CT) is broadly regarded as the reference NDT method for internal defect characterization and is paired with deep learning to automatically segment and measure the porosity and other internal defects (Acharya et al., 2022; Villarraga-Gómez et al., 2018; Zhu et al., 2020). This method is well suited for post-build qualification and certification of high-value, safety-critical metal or composite AM components where offline inspection time is acceptable and as the ground-truth reference for developing or validating ML models on faster inspection modalities (Acharya et al., 2022; Chen et al., 2022; Seifi et al., 2016; Villarraga-Gómez et al., 2018).

#### Advantages

Deep learning segmentation of CT slices substantially reduces the manual effort needed to quantify pore size distributions (Acharya et al., 2022; Zhu et al., 2020). Moreover, CT derived, voxel-level ground truth is frequently used to validate or train ML models built on faster *in-situ* modalities such as thermography or acoustic emission (Zhu et al., 2020).

#### Limitations

XCT requires longer time for scan and reconstruction. Moreover, the equipment cost, part-size constraints and radiation safety requirements make it impractical for *in-situ* inspection (Villarraga-Gómez et al., 2018). Also, data volumes for high-resolution volumetric scans are large, increasing computational and storage demands for the accompanying ML pipeline (Acharya et al., 2022; Zhu et al., 2020).

#### 5.2.5 Ultrasonic testing combined with ML

Ultrasonic inspection including conventional contact and non-contact laser ultrasound is employed to post-process or in-line inspection of larger metal AM/WAAM parts and geometrically complex components and in more recent work, combined with ML for automated defect classification (Davis et al., 2019; Lopez et al., 2018; Cerniglia and Montinaro, 2018; Lin et al., 2023).

#### Advantages

This method has good penetration depth in metals, enabling inspection of thicker sections than thermography or optical methods (Davis et al., 2019; Lopez et al., 2018). Laser ultrasound is fully non-contact and couplant-free, which is advantageous for the rough, geometrically complex surfaces typical of as-built AM parts (Davis et al., 2019; Cerniglia and Montinaro, 2018). Multiscale CNN or fuzzy neural network approaches applied to ultrasound images have improved automated quality classification of metal AM parts compared with manual signal interpretation (Lin et al., 2023).

#### Limitations

Signal interpretation in this method is complicated by rough surfaces, complex internal geometries and anisotropic AM microstructures which increase the burden on the ML model (Davis et al., 2019; Cerniglia and Montinaro, 2018). Resolution for very small pores is generally lower than X-ray CT. Additionally, labeled ultrasonic AM defect datasets remain limited compared with vision or thermal datasets (Lin et al., 2023).

#### 5.2.6 Acoustic emission (AE) combined with ML

Acoustic emission is most suitable as a complementary *in-situ* monitoring channel for L-PBF and DED processes particularly for early detection of crack initiation and keyhole-pore events, ideally fused with thermal or optical data rather than used alone.

#### Advantages

Sensors can be mounted externally to the build chamber, avoiding interference with the optical or thermal path used by other monitoring systems. Semi-supervised learning approaches are demonstrated to reduce the labeled data burden relative to fully supervised alternatives (Pandiyan et al., 2021).

#### Limitations

Acoustic signals from the process can mask defect-related emissions, giving a low signal-to-noise ratio (Pandiyan et al., 2021; Chen et al., 2023). Also, reliable defect localization from AE alone is difficult. It is typically used for detection/classification rather than precise sizing (Chen et al., 2023). Like thermography and ultrasound, published models are largely validated on single machines or materials, so cross-platform generalization is not yet established (Pandiyan et al., 2021).

#### 5.2.7 NDT and physics-informed machine learning (PIML)

PIML is best suited for NDT modalities where the underlying physics is well characterized and can be expressed in tractable governing equations specifically in thermographic monitoring, where heat conduction models can be embedded to predict porosity from surface temperature evolution and ultrasonic or acoustic based methods, where elastic wave propagation provides a natural physical constraint on defect induced signal changes (Farrag et al., 2025; Liu et al., 2021).

#### Advantages

One of the advantages of this combination is that PIML models can achieve reasonable accuracy on with less labeled dataset than purely data-driven models. It also improves generalizability as the model can be applied beyond the exact process parameters, machine or material system on which it is trained.

### Limitations

PIML requires high computational cost to incorporate partial differential equation-based physics constraints into the training loop and as the PIML applications are emerging, most of them are confined to single defect type, typically porosity and single processes such as L-PBF or FFF (Liu et al., 2021; Kapusuzoglu and Mahadevan, 2020).

### 5.3 Comparative summary

The Table 6 summarizes the advantages, limitations, and suitability of each ML-NDT combination discussed above.

**Table 6:** Comparative summary of advantages, limitations, and suitability of ML-NDT combination.

ML-NDT combination	Key advantages	Key limitations	Suitability
Infrared thermography + DL/ML	Real-time, fast, non- contact, cost-effective; *in-situ* capable; sensitive to sub-surface thermal anomalies	Emissivity/surface sensitivity; thermal blurring limits spatial resolution; heavy data/labeling needs	*In-situ* monitoring of L-PBF, DED and WAAM; Porosity, lack-of-fusion and crack propagation in AM components
Computer vision/ optical imaging + DL	Low-cost hardware, high frame rate, easy to automate	Surface defects only, cannot see internal defects	Layer-wise surface quality control, Polymer/FDM stringing and warping, ceramic surface cracks
X-ray computed tomography + DL	High resolution, strong ground truth for training other sensors	Slow, costly, offline only, radiation handling, huge data volumes	Post-build qualification/certification of high-value metal and composite parts
Ultrasonic testing + ML	Deep penetration in metals, Laser-UT is non-contact/ couplant-free	Complex signals from rough/ curved AM surfaces, coupling issues, lower resolution for small pores	Post-process inspection of large or geometrically complex metal AM or WAAM parts
Acoustic emission + ML	Continuous *in-situ* sensing; sensitive to crack initiation/ keyhole instability	Low signal-to noise ratio; Needs large labeled datasets	Real-time process monitoring of L-PBF/ DED for crack and keyhole pore prediction
NDT + PIML	Improve generalization, reduced dependence on labeled datasets	High computational cost, limited methodological maturity	Well characterized underlying physics of NDT modalities; Tractable governing equations

## 6 Discussion

This discussion synthesizes the findings presented in Sections 3–5. The trade-offs outlined below are drawn across studies that used different materials, AM processes, defect types, and validation protocols.

### 6.1 NDT techniques

The reviewed literature shows that defect detection in AM relies on five principal families of non-destructive testing (NDT) technique, applied either *in-situ* (during the build) or *ex-situ* (after the build is complete). The following findings have been derived from the reviewed literature.

- Infrared /Thermographic Monitoring is the most widely reported modality, spanning *in-situ* melt-pool and layer wise monitoring (Baumgartl et al., 2020; Ho et al., 2021; Klamert, 2025; Oster et al., 2024a,b; Chen et al., 2019; Zhang et al., 2020a,b, 2021, 2024) and *ex-situ* active and step heating thermography (Szymanik et al., 2021; Rodríguez-Martín et al., 2022; Szymanik, 2022; Boiko et al., 2022; Heifetz et al., 2020; Cerniglia and Montinaro, 2018; Montinaro et al., 2018).
- Computer Vision/Optical Surface imaging, a camera-based inspection of surface defects such as stringing, warping, and surface cracking is employed mostly for polymer and ceramic AM (Paraskevoudis et al., 2020; Zhou et al., 2024; Borish et al., 2020; Okarma and Fastowicz, 2019) whereas related confocal and scanning optical microscopy is used chiefly for surface and porosity metrology (Grimm et al., 2015; Wang et al., 2018).
- X-ray Computed tomography (CT) is used as the reference technique for volumetric, sub-surface defect characterization exclusively for *ex-situ* monitoring (Chen et al., 2021; Acharya et al., 2022; Villarraga-Gómez et al., 2018; Zhu et al., 2020).
- Ultrasound testing, including conventional contact ultrasound and non-contact laser ultrasound is applied to thicker or geometrically complex metal AM/WAAM parts (Cerniglia and Montinaro, 2018; Lopez et al., 2018; Davis et al., 2019; Lin et al., 2023).
- Acoustic emission which is continuous *in-situ* sensing of transient elastic-wave events is associated with crack initiation and keyhole instability (Pandiyan et al., 2021; Chen et al., 2023).

Across the reviewed set, *in-situ* techniques, predominantly thermography, and acoustic emission are favored when the objective is real-time process monitoring and control, whereas *ex-situ* techniques including CT, ultrasound and post-process thermography or vision are favored for detailed, quantitative post-build characterization, and certification (AbouelNour and Gupta, 2022; Chen et al., 2022; Seifi et al., 2016; Villarraga-Gómez et al., 2018; Everton et al., 2016). No single technique covers both objectives equally well, therefore AM defect detection studies increasingly combine more than one modality (Chicote et al., 2022; Bai et al., 2025; Saberironaghi et al., 2023).

### 6.2 ML/DL algorithms

The reviewed studies integrated a broad spectrum of ML and DL algorithms with the NDT techniques above and the choice of the algorithm family tend to track the structure of the underlying sensor data rather than the NDT modality itself.

- Convolutional Neural Networks (CNN) and their variants including CNN-AlexNet,3D-CNN, DID-CNN, DED-CNN, deep CNN (DCNN) and Region-based CNN (Res-CNN) are the dominant choices for image data such as thermographic frames, CT slices, and camera images and appear across nearly every NDT modality in Table 5 (Chen et al., 2019; Baumgartl et al., 2020; Paraskevoudis et al., 2020; Zhu et al., 2020; Chen et al., 2021; Ho et al., 2021; Szymanik et al., 2021; Acharya et al., 2022; Lin et al., 2023; Klamert, 2025).
- Recurrent and sequence models such as RNN, LSTM, and hybrid FCNN/LSTM architectures are applied where the signal is inherently time-dependent, such as acoustic emission streams or transient thermal responses (Chen et al., 2021; Boiko et al., 2022; Szymanik, 2022).
- Generative and anomaly-detection architectures including variational autoencoders (VAE) and GANomaly are used in semi-supervised or unsupervised settings where labeled defect examples are scarce, most notably for acoustic-emission-based L-PBF monitoring (Pandiyan et al., 2021).
- Classical ML algorithms including Random Forest, Support Vector Machine (SVM) and linear or Gaussian Regression are typically reserved for regression-style tasks such as defect sizing or property prediction rather than binary defect classification (Rodríguez-Martín et al., 2022; Oster et al., 2024b).
- Other specialized architectures include Fuzzy Neural Networks for ultrasonic image classification (Lin et al., 2023) and signal-decomposition algorithms including STSCS, STBSS, STSDL are developed specifically for pulsed thermography defect characterization (Heifetz et al., 2020; Zhang et al., 2024).

This pattern suggests that algorithm selection in AM defect detection is largely data-driven: image structured outputs such as thermal maps, CT slices, camera frames, attract CNN-family models, time-series sensor stream attract recurrent models, label-scarce problems attract generative or semi-supervised approaches and continuous valued targets such as defect size and mechanical property, attract classical regression methods (Pandiyan et al., 2021; Szymanik, 2022; Oster et al., 2024b; Bai et al., 2025).

### 6.3 Challenges in defect detection

Several recurring challenges emerge across the reviewed ML-NDT literature, spanning data availability and quality, model robustness, interpretability, and real-world deployment. These are discussed in detail.

#### 6.3.1 Limited labeled datasets and data quality

Ground truth defect labels in AM are expensive to obtain because they typically require destructive sectioning, CT scanning or expert visual or thermal annotation to confirm what a sensor signal actually corresponds to physical one (Villarraga-Gómez et al., 2018; Acharya et al., 2022; AbouelNour and Gupta, 2022). Consequently, the reviewed studies typically train on a few hundred to a few thousand labeled samples which is several orders of magnitude lesser than conventional computer vision benchmarks. Furthermore, some studies adopted semi-supervised or unsupervised strategies to reduce this dependency (Pandiyan et al., 2021; Zhang et al., 2021) but even these approaches rely on some labeled data for validation.

Data quality is a related and separate concern: raw sensor streams are affected by motion artifacts and lighting variation in optical images, frame loss in IR cameras and variability in ultrasonic signals. If these are not carefully filtered, such noise can be learned by the model as a feature rather than a true defect indicator (Szymanik et al., 2021; Zhou et al., 2024; Lopez et al., 2018; Szymanik, 2022). Because very few of the underlying datasets are published or shared, independent verification, reuse and pooling of data across studies remains severely limited (Bai et al., 2025; Everton et al., 2016; Saberironaghi et al., 2023).

#### 6.3.2 Model generalization and transferability across AM machines and materials

Nearly all reviewed models are trained and tested on data received from a single printer, a single material, and often a single build geometry (Klamert, 2025; Oster et al., 2024b). A model trained on one machine's thermal or acoustic signature may not transfer reliably to another machine even for the same alloy and process (Oster et al., 2024a,b) because of the differences between nominally identical AM machines in terms of laser power calibration, chamber atmosphere, and sensor placement or calibration etc.

Transferability is still harder across materials or across AM processes, since defect morphology and the underlying thermal, acoustic or optical signatures differ (Chen et al., 2019, 2023; Baumgartl et al., 2020). Few of the reviewed studies explicitly test cross-machine or cross-material transfer; most report single-domain train/test performance only, leaving real-world generalizability largely unverified (Zhang et al., 2020a; Pandiyan et al., 2021; Szymanik, 2022). This is a significant gap, since a model validated in one laboratory setting offers no guarantee of reliable performance when deployed on a different AM system in production.

#### 6.3.3 Explainability and interpretability (XAI)

Most of the reviewed deep learning models such as CNNs, RNN/LSTM networks, and generative architectures function largely as black boxes, producing a classification, probability score or predicted defect size without a rational for that output (Zhu et al., 2020; Chen et al., 2021; Ho et al., 2021). This is a meaningful barrier in AM quality assurance, particularly for safety-critical aerospace or medical components (Seifi et al., 2016; Chen et al., 2022). Only a small subset of the reviewed methods moves toward more interpretable outputs: physics-or simulation informed approaches such as Szymanik's numerically trained thermography model (Szymanik, 2022) and signal-decomposition algorithms such as those employed by Heifetz et al. (2020) provide some mechanistic grounding for their predictions, but systematic use of explainable-AI techniques is rare across the reviewed set.

#### 6.3.4 Industrial deployment

Moving an ML-NDT pipeline from a laboratory demonstration into production requires more than reported accuracy. None of the reviewed studies report a sustained, in-production deployment; all describe laboratory or pilot scale validation (Baumgartl et al., 2020; Pandiyan et al., 2021; Chen et al., 2023; Oster et al., 2024a). Broader industrial adoption is likely to depend on larger and standardized datasets, demonstrated cross-machine, and cross-material robustness, interpretable outputs suitable for audit trails and closer conformity with emerging AM-NDT standards such as ISO/ASTM TR 52905 (ASTM International, ISO/ASTM TR 52905, 2023).

Altogether, these challenges indicate that progress in ML-based NDT for AM depends less on introducing further novel algorithms and more on addressing data quality and availability, demonstrating generalization, and transferability across machines and materials, building interpretable and auditable models, and closing the gap between laboratory validation and industrial deployment (Everton et al., 2016; Bai et al., 2025; Saberironaghi et al., 2023).

## 7 Conclusion

This review has surveyed the growing body of work applying machine learning (ML) to non-destructive testing (NDT) and *in-situ* process monitoring of additively manufactured (AM) parts, spanning thermographic, ultrasonic, acoustic, optical, and X-ray computed tomography (CT) modalities. Among the modality-algorithm pairings surveyed, deep learning applied to *in-situ* thermographic monitoring of L-PBF process is the most mature combination. A substantial and consistent body of work has used convolutional neural networks (CNNs) and related architectures on melt-pool and layer-wise thermal images to detect porosity, cracking and other defect types with reported accuracies that are competitive with post-build inspection. Deep learning assisted X-ray computed tomography is the second most developed combination, particularly for internal porosity and void characterization in metal and polymer parts where CNN and U-Net segmentation models have been used to automate defect quantification that was previously performed manually. Machine vision based surface and geometric defect detection using conventional CNN classifiers is also comparatively mature, especially for extrusion-based and ceramic processes where surface anomalies such as stringing, warping, and layer delamination are visually distinctive and cameras are inexpensive to deploy. Acoustic emission and laser ultrasonic monitoring combined with ML classifiers represent an emerging but increasingly validated pairing for detecting cracks and keyhole pores during directed energy deposition and L-PBF. Applications of artificial neural networks to defect detection in fiber-reinforced polymer composites are more limited in volume but methodologically well established.

Despite this progress, several recurring limitations constrain the practical impact of the reviewed studies. First, most models are trained and validated on small, single-machine, single-material datasets which limits generalizability across printers, alloys polymers, and process parameters. Few studies have reported cross-machine or cross-material transfer results. Second, there is no widely adopted public benchmark dataset or evaluation protocol for AM defect detection which makes it difficult to compare reported accuracies across studies or to establish a genuine state of the art. Third, ground-truth labeling remains a bottleneck. A reliable defect annotation typically requires correlating *in-situ* signals with destructive sectioning or CT scans which is costly and slow. As a result, many datasets are small, imbalanced toward defect-free samples, or weakly labeled. Fourth, the large majority of published models are purely data-driven and offer little physical interpretability which is a barrier to their acceptance in a field where component qualification and certification depend on demonstrable, explainable evidence of soundness. Fifth, most studies are demonstrated under controlled laboratory conditions and stop short of real-time, closed-loop deployment, computational latency, sensor integration, and the practicalities of embedding inference within commercial control systems are rarely addressed in depth. Finally, the absence of standardized qualification frameworks that formally recognize ML-based NDT as an accepted inspection method means that, however accurate, these techniques currently sit outside the certification pathways used for critical AM components.

Several directions appear most likely to address these gaps. Since individual modalities are sensitive to only a subset of defect types, multimodal sensor fusion combining thermographic, acoustic, and optical signals within a single ML pipeline is a natural next step. This fusion could improve robustness and reduce false positive rates. Next, development and public release of standardized, multi-machine, multi-material benchmark datasets, and evaluation protocols would allow meaningful comparison between methods and accelerate progress in a way that isolated single laboratory studies cannot. Furthermore, transfer learning and domain adaptation techniques merit further attention to allow models trained on one printer or alloy system to be adapted efficiently to new hardware and materials with limited additional labeled data.

There is also a clear need for physics-informed and hybrid ML models that combine data-driven pattern recognition with known thermal, acoustic, or mechanical process physics, both to improve accuracy with less training data and to make model behavior more interpretable to certifying bodies. Relatedly, explainable AI methods should be developed specifically for AM-NDT applications so that noted defects can be traced back to physically meaningful signal features. On the deployment side, more research is needed on real-time, closed-loop systems that not only detect defects *in-situ* but feed that information back into process control to correct or halt a build, moving the field from passive inspection toward active quality assurance. Finally, closer collaboration between the ML-NDT research community and standards bodies is needed to define qualification and certification pathways that formally incorporate machine learning based inspection which will be a prerequisite for its adoption in safety-critical aerospace, medical, and structural AM applications.
